# Differential Differences in Methylation Status of Putative Imprinted Genes among Cloned Swine Genomes

**DOI:** 10.1371/journal.pone.0032812

**Published:** 2012-02-29

**Authors:** Chih-Jie Shen, Winston T. K. Cheng, Shinn-Chih Wu, Hsiao-Ling Chen, Tung-Chou Tsai, Shang-Hsun Yang, Chuan-Mu Chen

**Affiliations:** 1 Department of Life Sciences, and Agricultural Biotechnology Center, National Chung Hsing University, Taichung, Taiwan; 2 Department of Animal Science and Technology, National Taiwan University, Taipei, Taiwan; 3 Department of Animal Science and Biotechnology, Tunghai University, Taichung, Taiwan; 4 Department of Molecular Biotechnology, Da-Yeh University, Changhwa, Taiwan; 5 Department of Physiology, National Cheng Kung University, Tainan, Taiwan; The Babraham Institute, United Kingdom

## Abstract

DNA methylation is a major epigenetic modification in the mammalian genome that regulates crucial aspects of gene function. Mammalian cloning by somatic cell nuclear transfer (SCNT) often results in gestational or neonatal failure with only a small proportion of manipulated embryos producing live births. Many of the embryos that survive to term later succumb to a variety of abnormalities that are likely due to inappropriate epigenetic reprogramming. Aberrant methylation patterns of imprinted genes in cloned cattle and mice have been elucidated, but few reports have analyzed the cloned pig genome. Four surviving cloned sows that were created by ear fibroblast nuclear transfer, each with a different life span and multiple organ defects, such as heart defects and bone growth delay, were used as epigenetic study materials. First, we identified four putative differential methylation regions (DMR) of imprinted genes in the wild-type pig genome, including two maternally imprinted loci (*INS* and *IGF2*) and two paternally imprinted loci (*H19* and *IGF2R*). Aberrant DNA methylation, either hypermethylation or hypomethylation, commonly appeared in *H19* (45% of imprinted loci hypermethylated vs. 30% hypomethylated), *IGF2* (40% vs. 0%), *INS* (50% vs. 5%), and *IGF2R* (15% vs. 45%) in multiple tissues from these four cloned sows compared with wild-type pigs. Our data suggest that aberrant epigenetic modifications occur frequently in the genome of cloned swine. Even with successful production of cloned swine that avoid prenatal or postnatal death, the perturbation of methylation in imprinted genes still exists, which may be one of reason for their adult pathologies and short life. Understanding the aberrant pattern of gene imprinting would permit improvements in future cloning techniques.

## Introduction

Somatic cell nuclear transfer (SCNT) is the transmission of a differentiated somatic cell nucleus to an enucleated oocyte. SCNT is used to generate individuals with identical genetic backgrounds, increase the economic efficiency of animal preservation, produce transgenic animals, and cure genetic disorders or cancer [1], [2]. However, SCNT-cloned mammals usually have a low survival rate due to abortion, neonatal death and postnatal defects. Animals that have been successfully cloned during the past decade include sheep, cattle, goats, pigs, rabbits, mice, cats, and dogs [3], [4], [5]. Various types of somatic cell are used for transfer, including mammary gland epithelial, ovary epithelial, cumulus, granulosa, and ear fibroblast cells. Although a variety of nuclear cell types and stages of oocytes have been tried, the success rate still remains low [6]. Most of surviving clones have physiological problems; for example, large offspring syndrome (LOS) and placental abnormalities have been found in cloned cattle, sheep, and mice [7], [8], [9]. Notably, offspring that are produced by the natural mating of clones that have an abnormal phenomenon do not inherit the abnormality, providing evidence that precise, dynamic epigenetic control is a major requirement during the period of fertilization to blastocyst [10]. Therefore, understanding aberrant methylation patterns and correcting perturbed epigenetic modification will help improve the health of cloned animals.

Epigenetic reprogramming is an essential process in mammals to regulate DNA methylation and gene expression during gametogenesis and embryogenesis. This reprogramming could be performed by demethylases and DNA methyltransferases (Dnmts) to produce demethylated and methylated DNA, respectively. However, no demethylase has been identified in mammals [11], [12]. The loss of maternal nuclear Dnmt1 is the cause of aberrant methylation in imprinted genes during nuclear transfer [13]. The reprogramming process is divided into four parts: formation of primordial germ cells (PGCs), maturation of gametes, fertilization to produce a zygote, and embryonic stages. The methylation markers of imprinting are erased during the formation of PGCs and reestablished in the gamete genome. After fertilization, the non-imprinted methylation markers are demethylated, and then the methylation markers are reestablished during embryonic development. However, the methylated or non-methylated imprinting markers are maintained from the one-cell to the blastocyst stage [14]–[16]. This reprogramming process affects DNA methylation, chromatin histone acetylation, and other embryo growth mechanisms. During embryonic development, the methylation status of some imprinted genes is dynamic and has spatial and temporal requirements [17]. Accumulating evidence indicates that incomplete or inappropriate epigenetic modification of donor nuclei used for nuclear transfer is likely to be the primary cause of failure when cloning animals [18].

DNA methylation at cytosine residues within CpG dinucleotides is one common regulatory modification of gene expression. Differentially methylated regions (DMRs) are DNA regions with methylation differences between parental alleles. Some DMRs are also imprinting control regions (ICRs), which control several imprinted genes in a cluster [15]. Most imprinted genes contain DMRs, which are crucial in maintaining imprinting in mammalian genomes. Genomic imprinting is a uniparentally expressed pattern that includes many reading mechanisms: promoter methylation, antisense transcripts, boundaries, and silencers. It is also involved in the regulation of normal embryonic development, placental growth, parental-specific expression, X-chromosome inactivation, clustering effects of ICRs, and tissue-specific expression [19], [20]. A group of imprinted genes may encode signal transduction molecules, cell cycle regulators, transcription factors, enzymes, and non-coding RNAs [21]. So far, approximately 60 imprinted genes have been found in the human genome, but this number is expected to increase to at least 100 [22].

In general, the perturbation of mono-allelic expression of imprinted genes could cause the abortion of embryonic and fetal development during pregnancy. For example, *H19* and *insulin-like growth factor 2 (IGF2)* are well-studied imprinted loci with specific expression patterns controlled by the DMR of *H19*. CTCF-binding protein is also involved in *H19* downstream enhancer regulation [23]. The alteration of the imprinting status of an *IGF2* allele (loss of imprinting, LOI) results in biallelic expression during embryonic growth, whereas *IGF2* overexpression in mice causes prenatal or postnatal overgrowth that is similar to the symptoms of Beckwith-Wiedemann syndrome [24], [25].

In this study, four surviving cloned sows created by ear fibroblast nuclear transfer with whole-cell microinjection, each with a different life span and multiple organ defects, were used as samples in this epigenetic study analyzing the aberrant methylation of maternally and paternally imprinted loci. Four imprinted genes, *H19*, *IGF2*, *receptor of insulin-like growth factor 2 (IGF2R)*, and *insulin-1 (INS)*, were selected as targets to verify changes in the DMR methylation patterns of the cloned swine genomes compared with the wild-type genomes. Both *IGF2R* and *H19* are paternally imprinted, characteristically maternally expressed genes that encodes a growth-inhibitory factor and non-protein-coding RNA transcript of unknown function, respectively, whereas the other two genes, *IGF2* and *INS*, are maternally imprinted, characteristically paternally expressed genes that encode growth-promoting factors [26]. To quantify the CpG island methylation status in DMRs of each selected imprinted gene, we used Southern blot hybridization, methylation-specific PCR (MS-PCR), bisulfite sequencing, and a combined bisulfite and restriction assay (COBRA). Significant changes (either hypermethylation or hypomethylation) in the levels of epigenetic methylation were observed in the analyzed imprinted loci in different tissues of cloned sows. Here, we describe the characterization of these epigenetic changes in the examined tissues of cloned swine genomes.

## Materials and Methods

### Tissue sample collection of cloned and wild-type sows

Whole-cell intracytoplasmic microinjection of ear fibroblast cells was used to produce four surviving cloned piglets, named cloned pig Nos. 1 to 4 (CP1 to CP4), as described previously [27]. The sex of the cloned pigs was female, and the species of the enucleated oocytes, somatic cell donors, and recipient pigs were all of the Landrace breed. Three wild-type female pigs with the same genetic background and housed under the same husbandry conditions were used as normal controls. The control body weight was calculated as the average weight of normal newborn piglets from ten litters (98 piglets) of the Landrace breed of the same age as the cloned pigs. Animals were housed and handled according to the guidelines of the Animal Care Committee of the Animal Technology Institute Taiwan (ATIT approval ID: 93021). Physiological characteristics, including birth weight, life span, death weight, and major defects, were collected. Compared with wild-type piglets of the same age, the birth and death weights of all cloned piglets were significantly reduced. Tissues from all three germ layers of cloned pigs, including ectoderm-derived tissues (ear, brain), mesoderm-derived tissues (heart, muscle, kidney), and endoderm-derived tissues (liver, lung), and also extra-embryonic placenta were sampled. Tissues were separated into two parts for DNA and RNA extraction, snap-frozen in liquid nitrogen, and stored at −80°C until use [28].

### Isolation of genomic DNA

High-molecular-weight genomic DNAs were extracted by the proteinase K/SDS method as described in our previous report [29]. Briefly, 300 mg of each tissue was homogenized and resuspended in 230 µl lysis buffer (50 mM Tris-HCl, 100 mM NaCl, 100 mM EDTA, pH 8.0), followed by adding 400 µl lysis buffer, 70 µl 10% SDS, 10 µl RNase A (10 mg/ml) and 35 µl proteinase K (10 mg/ml). The tissue-digested reaction was mixed well and incubated at 55°C for 16 h. The reaction mixture was cleaned twice by adding equal volumes of phenol/chloroform (1∶1). The supernatant was transferred, 1.5× volume of pure ethanol was added to precipitate DNA, and the genomic DNA pellet was washed twice with 70% ethanol. The dried DNA pellet was then dissolved in 40 µl of distilled deionized water and stored at −20°C.

### Southern blot combined methylation-sensitive enzyme digestion

The pig *H19* and *IGF2* probes used for Southern blot hybridization were prepared by PCR amplification, with cloning and sequencing using the pGEM-T easy TA Cloning Kit (Promega, Madison, WI). The PCR primer sets were designed as follows: *H19* probe: 5′-GTGATCGGACTTCTGACCCT-3′ and 5′-TCTCCACACCCACAAGCCG-3′; *IGF2* DMR1 probe: 5′-AGGGACCTGCCGCTCTGCT-3′ and 5′-AGGACTGGGAAAG GAGAGGA-3′. To analyze the DNA methylation status in a specific locus, 10 µg of genomic DNA was completely digested with a CpG island cutter (*Pvu*II or *Nla*III) alone or together with a methylation-sensitive restriction enzyme (*Eag*I or *Bst*UI; New England Biolabs, Ipswich, MA) and then electrophoresed on 1.2% agarose gels. DNA was transferred onto Hybond N^+^ membranes (GE Healthcare, Piscataway, NJ) in 20× SSC [30]. Single-stranded sense and antisense probes were radio-labeled with α[^32^P]-dCTP using the Rediprime II random prime labeling system (GE Healthcare) as previously described [31]. Hybridizations were carried out overnight in a 42°C incubator, and membranes were washed according to our previous report [32]. After autoradiograph exposure, the image plate was scanned under a phosphoimager (Typhoon 9200; GE Healthcare).

### Methylation-specific PCR (MS-PCR)

Genomic DNA (0.5 µg) was treated with sodium bisulfite according to the manufacturer's recommendation (EZ DNA Methylation Kit™; Zymo Research Corp., Orange, CA) and amplified with specific primers for methylated or unmethylated DNA. All PCR reactions were performed on an ABI 2720 thermocycler (Applied Biosystems, Foster, CA) and in 25 µl volumes using the Platinum *Taq* DNA polymerase system (Invitrogen, Carlsbad, CA). PCR products were separated in 1.5% agarose gels. The M-set primers (*H19* M-set: 5′-TTTATTGTATTTTTGAACGGCG-3′ and 5′-CTAAAAACCGAAACG AACCCG-3′) contained at least three CpG sites to distinguish the methylation status of the investigated region. U-set primers (*H19* U-set: 5′- TTTTGAATGGTGTTGATGGTTTG-3′ and 5′-TAACCCATACTAAAAACCAAAACA-3′) overlapping the M-set primers were used to amplify the unmethylated region.

### Methylation analysis by COBRA

For the amplification of the pig *INS*, *IGF2* exon IX, and *IGF2R* intron II putative DMRs, PCR was performed using 10 ng of the bisulfite-converted genomic DNA as a template. The primer sets of COBRA-PCR were listed as follows: *IGF2* DMR2: 5′- GGGATAGGGGTTGGGGGGTTA-3′ and 5′-ATCTCAAAAAAAAAACCTAATAAAA AC-3′; *IGF2R* DMR: 5′-TTTTGTAGTAGTGTGAGATTTGG-3′ and 5′-TAACCTC ATACTTCCTAAAAACC-3′; *INS* DMR: 5′-TTGAAAGGGGTTAGTAGTAG-3′ and 5′- CTAAAAACCAAACTATCCCC-3′. COBRA-PCR products were purified with phenol/chloroform, followed by ethanol precipitation. The DNA was resuspended in 8.5 µl of distilled deionized water. Purified PCR products were then digested with 10 U of restriction enzymes (New England Biolabs) as follows: putative DMR products of *INS* and *IGF2* exon IX were digested with *Bst*UI at 60°C; putative DMR products of *IGF2R* intron II were digested with *HpyCH*4IV at 37°C. The products of these digestions were electrophoresed in a 6% native acrylamide gel, stained with 200 mg/ml ethidium bromide (EtBr; Sigma, St Louis, MO), and visualized and quantified using a Kodak 1D Image Analysis Software (Eastman Kodak, Rochester, NY).

### Bisulfite sequencing

To determine the methylation status of CpG sites within the *INS* of putative DMRs, primers were designed according to bisulfite standards (no CpG sites within primers) as described in the COBRA above. PCR reactions were performed in a total of 25 µl for each imprinted locus. Individual PCR products were purified with phenol/chloroform followed by ethanol precipitation. Purified PCR products were cloned into a pGEM T-easy Vector (Promega). Plasmid DNA was isolated using a Mini-M™ Plasmid Purification Kit (Viogene, Taipei, Taiwan) and sequenced using a BigDye Terminator cycle sequencing kit with an ABI PRISM 3100 DNA sequencer (Applied Biosystems).

### RNA isolation and semi-quantitative RT-PCR

Total RNA was isolated from homogenized tissues using TriReagent™ (Invitrogen) according to the manufacturer's recommendations. One microgram of total RNA was treated twice with 10 U RNase-free DNase I (Invitrogen) to degrade any contaminating DNA, and the reaction was halted by heat-inactivation. First-strand cDNAs were synthesized from 2 µg RNA with an oligo (dT) primer and MMLV reverse transcriptase (Promega) in a total volume of 25 µl [33]. RT-PCR was conducted using specific sets of primers for each imprinted gene analyzed: *H19*: 5′- ATTCTGGAGCCACTACACTACTTGA-3′ and 5′- AGGAGAGGAAAGAAGAGAAGA GAAAA-3′; *INS*: 5′- GGAGGCGCTGTACCTGGTGT-3′ and 5′- AGGGAACAGATGC TGGTGCAG-3′; *IGF2*: 5′- CTACTTTGGTGGCGACTGCTACT-3′ and 5′- GGGTGG TGGATAAAGAGGACGG-3′; *IGF2R* 5′- CTGCGAAGGAGAGGAGTACG-3′ and 5′′- TACCGGAGGGTCTGATTCTG-3′; *β-actin*: 5′- CATCACCATCGGCAACGA-3′ and 5′- TTCCTGATGTCCACGTCGC-3′. The mRNA expression levels of each imprinted gene present in the cloned and normal swine genomes were quantitatively measured by Kodak 1D densitometer software and normalized to the level of *β-actin* mRNA expression [29].

### The analysis and quantification of methylation changes

The definition of hypermethylation or hypomethylation (±10% compared to the wild-type tissues) was as previously published in an analysis of the methylation changes of human cancer [34]. The methylation percentage of the *H19* putative DMR was calculated according to the Southern blot and MS-PCR data. Southern blotting bands were quantified as previously described [35]. The following formula was used to calculate the methylation percentage from MS-PCR results used: (intensity of M-set band)/(intensity of M-set band + intensity of U-set band)×100 (%). The methylation percentages of *INS*, *IGF2*, and *IGF2R* were based on the COBRA data. The quantification method of COBRA was as previously published [36].

## Results

### Identification of putative DMRs of imprinted genes in the swine genome

**Figure 1:**
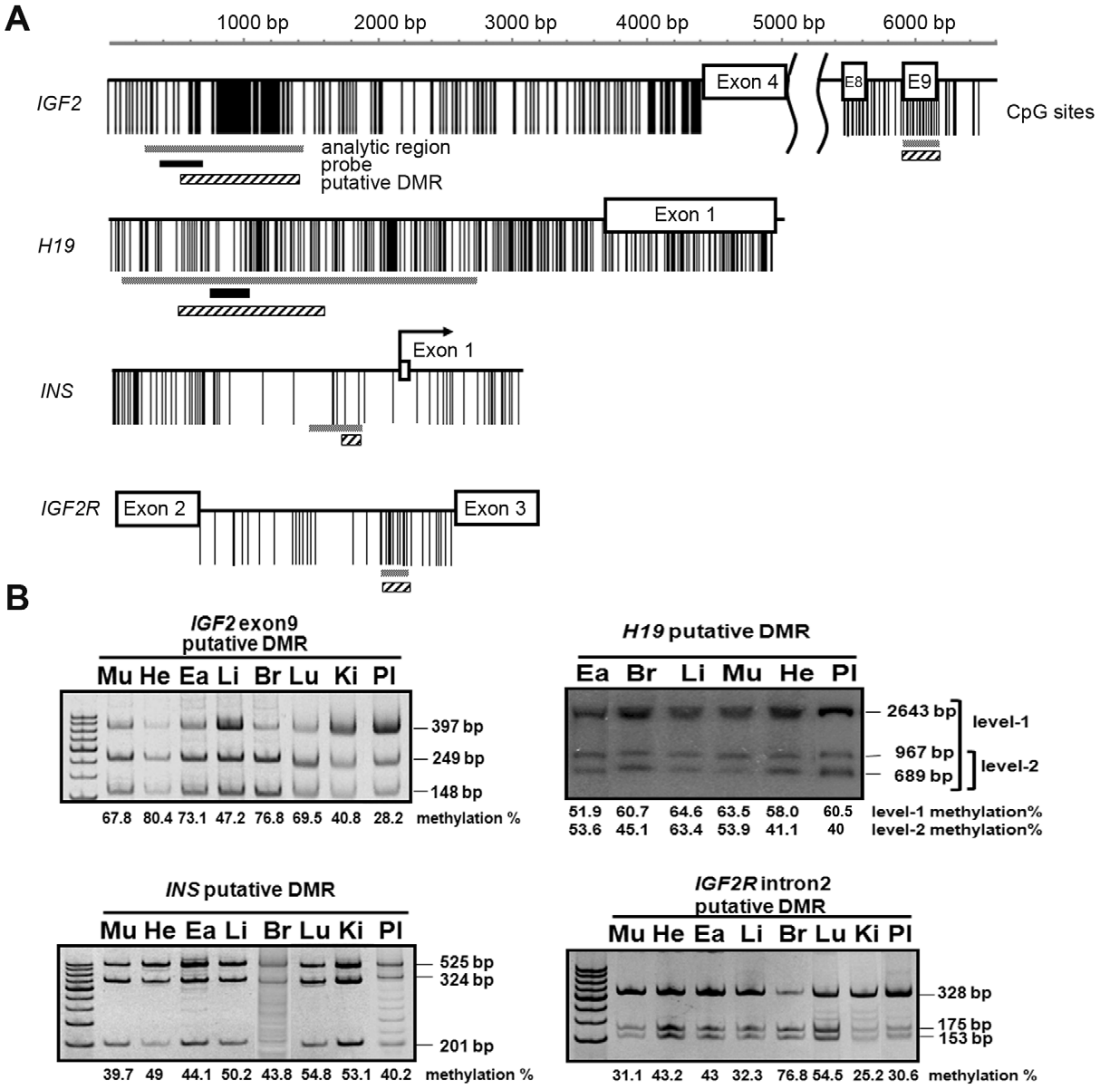
Identification of the putative DMRs of four imprinted genes and their normal differential methylation patterns in the different tissues of wild-type pigs. (A) Schematic of CpG site distributions in the putative DMRs of four imprinted genes, *IGF2*, *H19*, *INS*, and *IGF2R*. Vertical black lines represent each CpG site. Horizontal gray bars represent analyzed regions. Horizontal reticular bars represent putative DMRs in the imprinted genes. Horizontal solid black bars represent probes used for Southern blot hybridization. The upper line indicates the scale bar for DNA length. The putative DMR of *H19* is located between nt 30,856 and nt 33,489 (GenBank accession no. AY044827). The CpG island of *H19* corresponds to our designed probe, which ranges from nt 31,411 to nt 31,818. The putative DMR1 of pig *IGF2* is located between exon 3 and exon 4 and ranges from nt 17,620 to nt 18,796 (GenBank accession no. AY044828). The CpG island of *IGF2* corresponds to our designed probe, which ranges from nt 17,733 to nt 18,048. The putative DMR2 of *IGF2* is located in exon 9, nt 27,441 to nt 27,819 (GenBank accession no. AY242102.1). The putative DMR of pig *INS* is located between nt 1,456 and nt 2,323, and the probe ranges from nt 1,663 to nt 1,986 (GenBank accession no. AY242112). The putative DMR of *IGF2R* is located between exon 2 and exon 3 (GenBank accession no. AF339885). (B) The normal differential methylation patterns of the four imprinted genes in several tissues of wild-type pigs. The methylation statuses of *IGF2*, *INS*, and *IGF2R* were assayed by COBRA. The methylation status of *H19* was assayed with Southern blot analysis. The numbers under the images indicate the average methylation percentage in the different tissues of three wild-type pigs (n = 3). Mu: muscle; He: heart; Ea: ear; Li: liver; Lu: lung; Ki: kidney; Br: brain; Pl: placenta.

The DMRs of imprinted genes have regions that are highly conserved across various mammal species. First, we compared the well-known DMRs of *IGF2*, *H19*, *INS*, and *IGF2R* in the human, bovine, and mouse genomes to identify the counterparts of these DMRs in pig genome. The putative DMR1 of the pig *IGF2* gene was predicted to be located in intron 3, and the putative DMR2 of pig *IGF2* was in exon 9 (Figure 1A). The putative DMR of the pig *H19* gene was located upstream of the promoter, between −2856 and −1489 nucleotides (nt). We used the online software MethyPrimer with restrictive conditions (GC percent >50.0%, CpG observe/expect >0.6) to identify the distribution of CpG islands. The putative DMR of the pig *INS* gene was located before exon 1. Previously, there was no pig *IGF2R* intron sequence available in GenBank. The entire second intron of the pig *IGF2R* gene was newly cloned and sequenced (GenBank accession no. GQ888762) in this study. It contained a putative DMR based on comparisons with other species (Figure 1A). We further demonstrated that these selected regions of the four imprinted genes exhibited potentially differential methylation patterns in various tissues from wild-type pigs via Southern blot, MS-PCR, and COBRA (Figure 1B). The four putative DMRs in these imprinted genes were used to estimate the methylation perturbations in cloned pig genomes.

### Aberrant methylation of the *H19* gene in various tissues from cloned pigs

**Figure 2:**
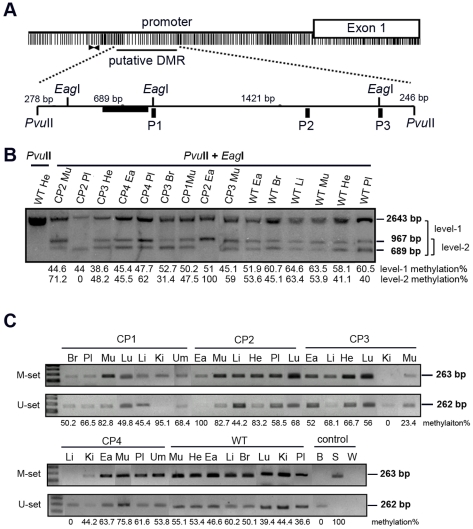
Methylation status of the *H19* putative DMR in cloned and wild-type pigs. (A) Schematic of the putative DMR of *H19*, located in the promoter. The Southern blot hybridization probe is shown as a black box. P1, P2 and P3 indicate three CTCF-binding sites of the putative DMR. (B) Southern blot hybridization results in the *H19* DMR in cloned pigs. The level-1 methylation percentage was calculated by the bands of 2643 bp, 967 bp, and 689 bp. The level-2 methylation percentage was calculated by the bands of 967 bp and 689 bp. (C) Methylation-specific PCR analysis of the *H19* promoter region in cloned pigs. The black arrow shown in Figure 2A indicates the primer sets used in the MS-PCR assay. The number below the panel indicates the methylation percentage. Br: brain; Ea: ear; He: heart; Ki: kidney; Li: liver; Lu: lung; Mu: muscle; Pl: placenta; Um: umbilical cord; B: blood; S: blood treated with *Sss*I; W: ddH_2_O.

The *H19* gene is a classical maternally expressed imprinted gene, and the aberrant methylation of the *H19* DMR often occurs in genetic diseases, growth retardation, prenatal lethality, and many kinds of cancer [37], [38]. We examined pig genomic DNA to determine whether an aberrant methylation pattern of the *H19* putative DMR occurred in adult cloned pigs compared with wild-type pigs (Figure 2B). Southern blot analysis indicated that the *H19* DMR in wild-type pigs showed three methylation patterns: a full methylation pattern (2.6 kb band), a partial methylation pattern (967 bp band), and an unmethylated pattern (689 bp band) (Figure 2B). Our data suggest that the CpG methylation of the first *Eag*I restriction site (near the CTCF1-binding site of *H19*) exists as a unique methylation pattern of the cloned pig genome. However, extremely aberrant methylation of *H19* DMR observed in the cloned pig genomes, including the lack of a 967 bp band in the placenta (Pl) and a 689 bp band in the ear (Ea) of CP2 (arrows in Figure 2B). We further categorized the extent of methylation into two levels. Level-1 was determined by three probed bands (2643 bp, 967 bp, and 689 bp). Level-2 was determined by two probed bands (967 bp and 689 bp). In the level-1 methylation, CP1 muscle, CP2 muscle, CP2 placenta, and CP3 heart showed hypomethylation patterns. In the level-2 methylation, CP2 muscle, CP2 ear, and CP4 placenta showed hypermethylation patterns, but CP2 placenta and CP3 brain showed hypomethylation patterns. Furthermore, we designed a primer set for MS-PCR upstream of the first *Eag*I (black arrowheads in Figure 2A) to confirm the methylation status in the *H19* promoter region. The wild-type (WT) control panel showed a normal methylation pattern in this region; however, various tissues from cloned pigs exhibited aberrant methylation statuses compared to the wild-type pig. CP1 kidney, CP2 ear, CP3 liver, CP3 kidney, and CP4 liver showed extremely aberrant methylation in the putative DMR of *H19* (Figure 2C; also see Table S1).

### Aberrant methylation of the *IGF2* gene in various tissues from cloned pigs

**Figure 3:**
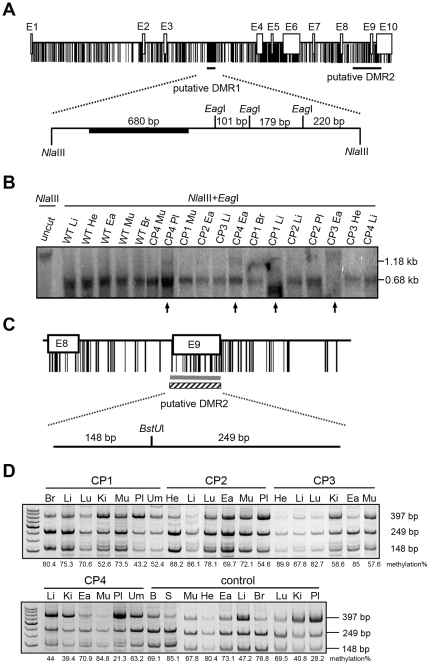
Methylation status of the putative *IGF2* DMR in cloned and wild-type pigs. (A) Schematic of the location of the putative DMR1 and DMR2 in the *IGF2* gene. Southern blot hybridization was performed with a probe, shown as a black box. E indicates the exon, and the reticular black line indicates CpG sites. (B) Southern blot hybridization results for the *IGF2* putative DMR1 in cloned pigs. Arrows indicate slight methylation in cloned pig tissues. (C) Schematic representation of the restriction enzyme site and the length of the COBRA product at the *IGF2* putative DMR2. Horizontal reticular line indicates the analytic region. Horizontal candy-striped line indicates the putative DMR2. (D) COBRA analysis of the *IGF2* putative DMR2 of exon 9 in cloned pigs and wild-type pigs. Methylation percentages are shown below the panel. The numbers under the figure in the control panel indicate the average methylation percentage of three wild-type pigs. Br: brain; Ea: ear; He: heart; Ki: kidney; Li: liver; Lu: lung; Mu: muscle; Pl: placenta; Um: umbilical cord; B: blood; S: blood treated with *Sss*I; W: ddH_2_O.

The *IGF2* gene is paternally expressed and located upstream of the *H19* gene. The methylation status of the *H19* DMR can concurrently affect the *IGF2* DMRs. The putative DMR1 of the pig *IGF2* gene was chosen as a target. It was estimated to be located in intron 3 (Figure 3A) based on the human, mouse, and cattle genomes. Southern blot combined with a methylation-sensitive enzyme assay showed that the putative *IGF2* DMR1 from wild-type pigs exhibited an unmethylated status, whereas several cloned pig tissues, such as CP1 liver, CP3 ear, CP4 placenta, and CP4 ear, displayed a slight methylation pattern (Figure 3B). The hybridized signal at 1.18 kb indicates full methylation and the band at 0.68 kb indicates the absence of methylation. The second putative DMR of *IGF2* (DMR2), which was located between exon 8 and exon 9, was further analyzed (Figure 3C). COBRA showed that this putative *IGF2* DMR was differentially methylated in wild-type pig tissues (Figure 3D). The methylation score (%) of the COBRA data were calculated as described previously [34], and any *IGF2* DMR2 in cloned pig tissues containing more than ±10% methylation changes compared with the wild-type pig was considered aberrantly methylated (Figure 3D). For example, liver tissues of CP1, CP2, and CP3 showed a hypermethylated pattern in the *IGF2* putative DMR2 region, but CP4 liver did not. Taking together all of the examined tissues from the cloned pigs except the placenta and umbilical cord, 40% showed a hypermethylated *IGF2* pattern, 0% showed a hypomethylated pattern, and 60% showed a normal methylation pattern in the testing sample size (see Tables S2 and S5).

### Aberrant methylation of the *INS* gene in various tissues from cloned pigs

**Figure 4:**
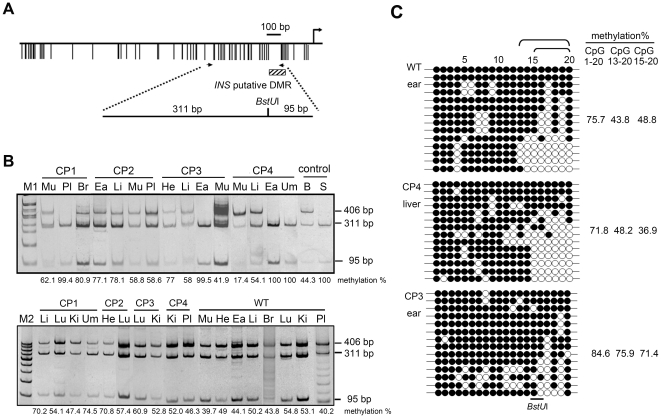
Dissection of the methylation status of the putative *INS* DMR in cloned and wild-type pigs. (A) A schematic diagram of the pig *INS* gene showing the relative positions of promoter, exon 1 and CpG islands. The black arrow indicates the location of the primer set. The striped box indicates the putative DMR. The horizontal line indicates the CpG site. The PCR product was digested with *Bst*UI. (B) The putative DMR of the maternally imprinted *INS* gene in cloned pigs and wild-type pigs was analyzed by COBRA. The numbers under the figure in the WT panel indicate the average methylation percentage of three wild-type pigs. (C) Bisulfite sequencing of the *INS* putative DMR in WT ear, CP4 liver, and CP3 ear. Open and closed circles indicate unmethylated and methylated CpG sites, respectively. The top number indicates the CpG site position of the analyzed *INS* putative DMR. The bottom line indicates the *Bst*UI recognition site. Fourteen clones of each tissue were sequenced. The methylation percentage calculations were divided into three parts: CpG sites 1 to 20 (all), CpG sites 15 to 20, and CpG sites 13 to 20. For example, WT ear showed a total methylation percentage of 75.7 (212 methylated CpG sites/20 CpG sites ×14 clones = 75.7%). M1: Bio 100 DNA ladder; M2: 500 bp DNA ladder; Br: brain; Ea: ear; He: heart; Ki: kidney; Li: liver; Lu: lung; Mu: muscle; Pl: placenta; Um: umbilical cord; B: blood; S: blood treated with *Sss*I; W: ddH_2_O.

The *INS* gene is a paternally expressed gene. The putative pig *INS* DMR is located in the promoter region (Figure 4A). The methylation status of the *INS* putative DMR in cloned pigs was analyzed by COBRA-PCR and *Bst*UI digestion. In this study, the definition of normal methylation was based on the methylation status of the *INS* putative DMR in wild-type pig tissues (Figures 1B and 4B). Several cloned pig tissues, including CP1 placenta, CP3 ear, CP4 ear, and CP4 umbilical cord, showed almost complete methylation of this putative *INS* DMR (Figure 4B; also see Table S3). Subsequently, bisulfite sequencing was performed to confirm the methylation pattern of these aberrant regions in the above tissues. The overall methylation percentage of CpG sites 1–20 in wild-type ear, CP4 liver, and CP3 ear were 75.7%, 71.8%, and 84.6%, respectively. Methylation percentage of the CpG sites 13–20 in wild-type ear, CP4 liver, and CP3 ear was 43.8%, 48.2%, and 75.9%, respectively. Moreover, the methylation percentage of CpG sites 15–20 in wild-type ear, CP4 liver, and CP3 ear was 48.8%, 36.9%, and 71.4%, respectively (Figure 4C). The *INS* putative DMR had a significantly hypermethylated pattern in CP3 ear tissue when compared with wild-type. Taking together all of the examined tissues from the cloned pigs except the placenta and umbilical cord, 50% showed a hypermethylated *INS* pattern, 5% showed a hypomethylated pattern, and 45% showed a normal methylation pattern in the testing sample size (see Table S5).

### Aberrant methylation of the *IGF2R* gene in various tissues from cloned pigs

**Figure 5:**
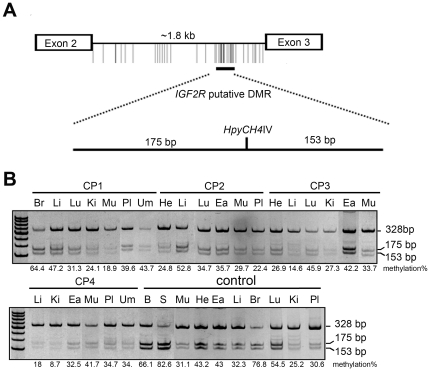
Methylation status of the *IGF2R* putative DMR of intron 2 in cloned pigs and wild-type pigs by COBRA analysis. (A) Schematic diagram showing the distribution of CpG sites of the pig *IGF2R* gene. (B) COBRA data showing the methylation status of the *IGF2R* intron 2 putative DMR in control and wild-type pigs. The numbers under the figure in the control panel indicate the average methylation percentage of three wild-type pigs. Br: brain; Ea: ear; He: heart; Ki: kidney; Li: liver; Lu: lung; Mu: muscle; Pl: placenta; Um: umbilical cord; B: blood; S: blood treated with *Sss*I; W: ddH_2_O.

Various tissues of cloned porcine were investigated for their methylation status at the putative DMR within *IGF2R* intron 2 (Figure 5A). The control panel showed a differentially methylated status, ranging from 25.2% to 76.8% in wild-type tissues analyzed by COBRA (Figure 5B). Aberrant methylation patterns were observed in the *IGF2R* putative DMR of cloned pigs; for example, the livers of CP1 and CP2 showed a hypermethylated pattern, while a hypomethylated pattern existed in CP3 and CP4 (Figure 5B, also see Table S4). Taking together all of the examined tissues from cloned pigs except the placenta and umbilical cord, 15% showed a hypermethylated *IGF2R* pattern, 45% showed a hypomethylated pattern, and 40% showed a normal methylation pattern in the testing sample size (see Table S5).

### Effects of DNA methylation on the expression of the analyzed genes

**Figure 6:**
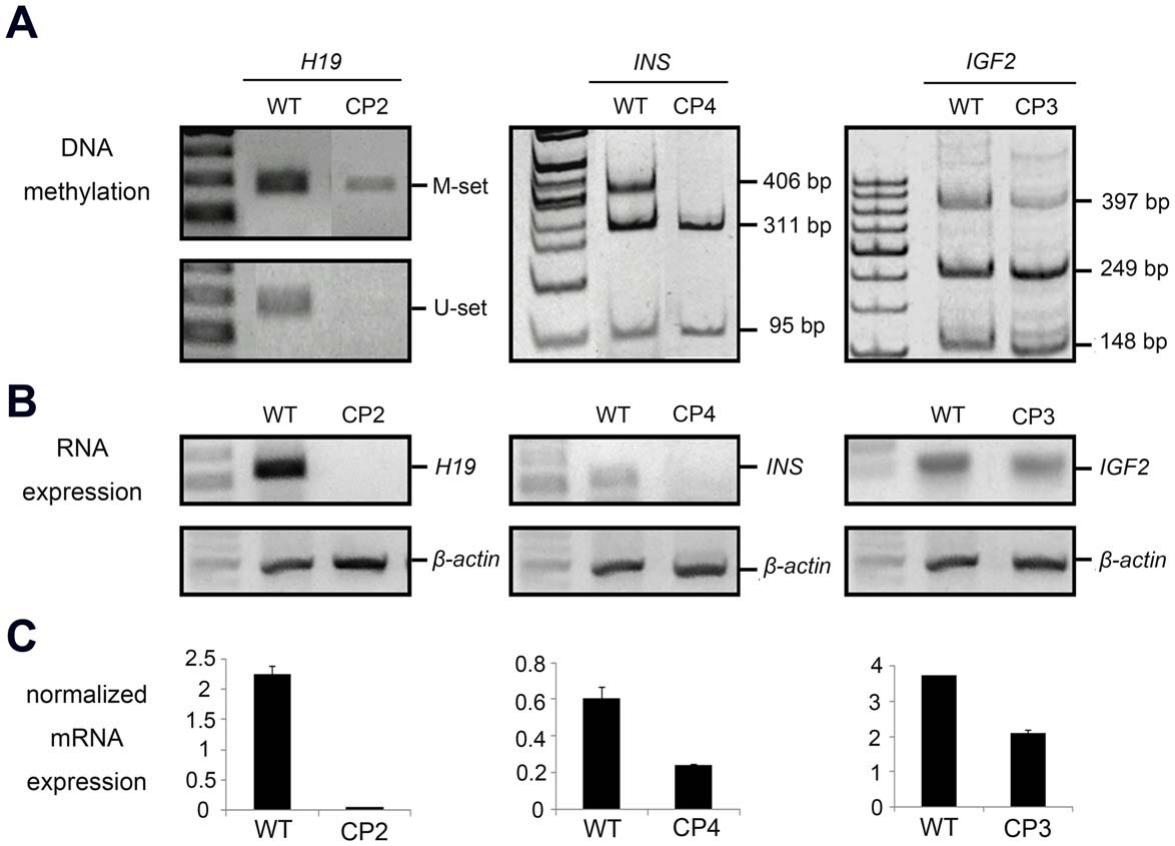
The aberrant methylation of the *H19*, *INS* and *IGF2* genes and their mRNA expression levels in cloned pigs. (A) DNA methylation statuses of *H19*, *INS*, and *IGF2* in CP2 ear, CP4 ear, and CP3 ear, respectively. (B) mRNA expression levels of *H19*, *INS* and *IGF2* gene in CP2 ear, CP4 ear, and CP3 ear, respectively. (C) mRNA levels from panel B relative to *β-actin*.

Semi-quantitative RT-PCR was used to verify the aberrant expression status of the four analysed genes. For the putative DMR of the *H19* gene, hypermethylation was shown in CP2 ear tissue (Figure 6A). Thus, the mRNA expression of *H19* was absent in the ear tissue of CP2 (Figure 6B and 6C). Similar results were found for the putative *INS* DMR in CP4 ear tissue as well as in the putative *IGF2* DMR2 in CP3 ear tissue (Figure 6B and 6C). To validate the cause–effect relationship of putative DMR hypermethylation in the downregulation of imprinted genes, a demethylation study was performed. Treatment of pig ear fibroblasts with different concentrations (0.5–2.0 µM) of a demethylating agent, 5-aza-2′-deoxycytidine (5-aza-dc), resulted in a reduction of the methylation levels of the *IGF2*, *H19*, and *INS* putative DMRs, thereby restoring the mRNA expression of these imprinted genes (see Figure S1).

## Discussion

Our data reveal that cloned pigs exhibit widespread defects in the methylation of imprinted genes, in addition to morphological abnormalities. All analyzed samples of CP2 and CP4 showed aberrant methylation patterns in the *H19* and *IGF2R* putative DMRs, respectively (Table 1 and Table S6). The aberrant methylation may occur during early embryonic development. We also found that aberrant methylation existed even though the cloned pigs grew into adult animals. These results indicate that the SCNT cloning process may disrupt the normal epigenetic reprogramming during embryogenesis but that some types of mechanisms are still maintained during normal fetal development. The mRNA expression levels of imprinted or non-imprinted genes can also be affected. The cloned pigs died naturally and had a shorter life-span than wild-type pigs. In these specific cloned pigs, physiological abnormalities occurred frequently in heart and bone (see Figure S2).

**Table 1:** Body weight, survival time, and aberrant methylation status of imprinted genes in wild-type and of cloned pigs.

Cloned pig ID	Weight at birth^1^ (kg)			Survival time(day)	Weight at death (kg)			Aberrant methylation statuses (%)						
	NT piglet^a^	WT^*b^	(a–b)/b(%)		NT pig^c^	Same age WT^d^	(c–d)/d (%)	Total	P	M	*H19*	*IGF2R*	*IGF2*	*INS*
CP1	1.3	1.5	−13.3	171	53.5	88.5	−39.5	55	60	50	60	60	40	60
CP2	0.87	1.5	−42	355	124.1	179	−30.7	65	80	50	100	60	20	80
CP3	1.17	1.5	−22	195	60.5	85	−28.8	65	58	50	83	33	67	33
CP4	1.34	1.5	−10.6	3	1.1	1.6	−31.3	56	75	25	50	100	25	50

1 The body weight of control newborn piglets is an average value from 10 litters (98 piglets) of same-age piglets of the nuclear donor pig breed. WT: wild-type. M: maternally imprinted genes (*H19* and *IGF2R*). P: paternally imprinted genes (*IGF2* and *INS*).

The cloned pigs used in this experiment exhibited a lower birth weight than wild-type pigs (Table 1). CP1 exhibited the oval foramen phenomenon and delay in the development of the limb skeleton. A significant reduction in birth weight was observed in CP2, which also suffered from gastric ulcer, peritonitis, and pericarditis, whereas CP3 showed a valvular disease and fibrosis of the heart. CP4 showed pneumonia and scoliosis. The SCNT processes in cloned pigs may cause the loss of regulation in gene expression or aberrant organ development. As the results of our methylation study suggest, the putative DMR of *H19* was dramatically modified in CP2 ear and placenta (Figure 2A). The birth weight of all four cloned pigs was reduced. CP2 showed the lowest birth weight (42% body weight lower than wild-type), and the degree of aberrant methylation in CP2 was higher in the paternally imprinted genes (80%) than in the maternally imprinted genes (50%) (Table 1). The opposite aberrant methylation pattern was presented in the other three cloned pigs. These results may be associated with the fact that paternally imprinted genes tend to inhibit embryonic growth, while the maternally imprinted genes tend to promote embryonic growth [39].

The cytoplasm of enucleated oocytes may possess the critical factors that control the normal reprogramming process [40]. Whereas incomplete demethylation changes are observed in cloned cattle [41], demethylation occurs more frequently in cloned pigs than in other species [42]. Previous studies have examined the methylation statuses of repetitive sequences in the cloned pig genome. The typical demethylation pattern is observed in the 2-cell through the blastocyst stage of porcine embryo development [42]. Wei et al. [13] also confirmed that demethylation status occurs in the first two cell cycles of the cloned porcine due to the lack of Dnmt1 in the oocyte, which affects the methylation of *H19* and *IGF2*. Kang *et al*. [41] observed that the satellite genes of NT bovines were hypermethylated. During DNA methylation reprogramming, the methylation statuses of NT sheep are higher than *in vivo* embryos from the 2-cell to the 16-cell stage [43]. However, to date, there are few studies on the aberrant methylation of imprinted genes in cloned pigs. We propose that the typical demethylation pattern of the cloned pig genome would affect the methylation pattern in the imprinted genes. Repetitive sequences maintain the donor-type methylation status in cloned rabbit and bovine embryos. Thus, the cause of demethylation in cloned embryo genomes seems to be determined by the recipient oocyte but not by the donor cell [44]. This finding suggests that the mechanisms involved in epigenetic reprogramming are species-specific. As expected, the reduction of body weight in this study agreed with the results of previous reports [6], [18]. Different strains of mouse oocytes with different epigenetic inheritance show differential cloning efficiency [40], which suggests that the factors that exist in oocytes may be critical to maintaining proper reprogramming processes during the embryonic development of cloned animals. Recent reports also suggest that the factors in the normal fibroblast cytosol can restore the aberrant imprinting status of tumor cells to the normal pattern [45]. Therefore, the procedure of nuclear transfer may prevent the accumulation or activity of factors that regulate the normal reprogramming process.

The methylation status of mouse and human *H19* DMRs shows a semi-methylation pattern. Interestingly, a unique methylation pattern of the initial CpG sites of the *H19* putative DMR existed in cloned pig genomes near the CTCF1 P1 site (Figure 2B). The CTCF binding sites (P1, P2, and P3) in the *H19* DMR are sensitive to methylation changes in cloned porcine genomes or human cancers [46], [47]. The aberrant pattern observed in cloned porcine showed either hyper- or hypo- methylation in the *H19* CTCF3 binding site, but there was no significant change in methylation status in the CTCF1- or CTCF2- binding sites [44]. Aberrant methylation of DMRs results in the loss of regulation of imprinted genes [48]–[50]. Therefore, the reprogramming process may be disrupted by the nuclear transfer technique, which may lead to LOI in the *H19* gene. Moreover, mRNA expression of other genes that are controlled by events downstream of *H19* may become dysregulated in a normal expression pattern [31]. Here, the aberrant methylation pattern in cloned pigs frequently appeared near the CTCF1 P1 site of the *H19* DMR.

*IGF2* and *H19* are reciprocally imprinted genes in a boundary regulation phenomenon. The imprinting pattern (uniparental RNA expression and DMR characteristics) of *IGF2* and *H19* has also been confirmed in the porcine genome [51], [52]. Based on Southern blotting, the putative DMR1 region of pig *IGF2* showed no methylation in wild-type pig tissues (Figure 3B). However, the CpG sites of exon 9 in the *IGF2* putative DMR2 showed a differential methylation phenomenon (Figure 3D). In the *IGF2* putative DMR1, a few tissues of cloned pigs (CP4 placenta and ear, CP1 liver, and CP3 ear) showed some methylation. In the *IGF2* putative DMR2, various tissues exhibited abnormal methylation in cloned pigs when compared with wild-type. These data indicate that DMR2, but not DMR1, exhibits a differential methylation phenomenon and easily acquires aberrant methylation in cloned pigs. These data agreed with previous findings that the putative DMRs of pig *H19* and *IGF2* exhibit a specific parental methylation at the 2-cell and blastocyst stages [53], [46].

The presence of a variable-number tandem repeat (VNTR) in the 5′ region of the *INS* promoter suggests that *INS* is potentially an imprinted gene, and its imprinting status could be associated with insulin-dependent diabetes mellitus [54]. The paternal expression pattern of *INS* has also been found in the yolk sac of humans and mice [55], [56]. The chromosomal location of *INS* is the same in the porcine, human, and mouse genome, i.e., upstream of *IGF2* [57], [58]. The promoter of the porcine *INS* gene had high-density CpG sites located just upstream of exon 1 (Figure 4A). By COBRA, this *INS* putative DMR was identified to contain a differential methylation pattern in the wild-type pig genome (Figure 1B). Either hypermethylation or hypomethylation of this *INS* putative DMR occurred in the cloned pig tissues (Table S7). The precise location of the DMR was further confirmed by bisulfite sequencing, which showed that it existed in CpG sites 15 through 20 (Figure 4C).

*IGF2R* and *IGF2* are imprinted genes with opposing functions [59]. These genes are also reciprocally regulated during fetal growth. Loss of *IGF2R* imprinting correlates with LOS in sheep [50], [60]. We used comparative sequencing analysis to define the organization of the pig, mouse, bovine, and human *IGF2R* putative DMRs and found that it was located in intron 2 of pig *IGF2R* gene (Figure 5A). A normal differentially methylated pattern was shown in the *IGF2R* putative DMR of wild-type pig tissues. However, a largely hypomethylated phenomenon was detected in the tissues of cloned pigs (Tables S7 and S8).

In this study, the four cloned pigs we studied had many defects, especially in the heart, lung, and gastric tissues (Table 1). These defects may have been associated with the loss of regulation of DNA methylation or mRNA expression during embryonic development [45], [61]. The delay in limb bone growth in CP1 may also have resulted from the NT process. A previous study of cloned pigs also showed abnormal development in the elbow joint bones [62]. Three hypermethylated tissue samples, CP2 ear (in the *H19* putative DMR), CP4 ear (in the *INS* putative DMR), and CP3 ear (in the *IGF2* putative DMR), were tested for the mRNA expression of the hypermethylated genes. Low expression levels of these imprinted genes were consistently observed in cloned pigs compared with the wild-type pigs (Figure 6).

Aberrant DNA methylation induced obvious abnormalities in our SCNT-derived embryos and their offspring. The abnormal phenomenon in CP2 and CP3 included an enlarged tongue (macroglossia), which is similar to Beckwith-Wiedemann syndrome, and is caused by aberrant methylation and expression of *IGF2* in humans [63]. Furthermore, the enlargement of the right ventricle in cloned pigs has also been reported [6]. CP3 and CP4 also suffered from this heart defect.

In conclusion, we confirmed that the putative DMRs of *H19*, *IGF2*, *INS*, and *IGF2R* in the wild-type porcine genome show differential methylation patterns. Specific proportions of epigenetic aberrations, either hypermethylation or hypomethylation, were observed in the adult tissues of the examined cloned pigs. These data will help further our understanding of the importance of imprinted genes during the development of normal or cloned swine and contribute to improvements in cloning techniques.

## Supporting Information

Figure S1 **Changes in the methylation of putative DMRs and in the mRNA expression of four imprinted genes after treatment of pig ear fibroblasts with 5-aza-dc for 48 h.** (A) The methylation percentage was quantified by COBRA. The methylation statuses of four imprinted genes (*IGF2*, *H19*, *INS*, and *IGF2R*) at their putative DMRs were decreased after different concentrations of 5-aza-dc treatment. (B) The mRNA expression was normalized to *β-actin* after real-time qRT-PCR. Three genes (*IGF2*, *H19*, and *IGF2R*) had increased mRNA expression after treatment with 0.5 µM 5-aza-dc. In contrast, the mRNA expression of *INS* significantly increased after treatment with 1.5 µM 5-aza-dc. The 5-aza-dc experiments were performed in 6-cm dishes seeded with 1.2×10^5^ pig fibroblasts in DMEM. All experiments were performed three times, and the data are expressed as the means ± SDs; *p<0.05, **p<0.01.
(TIF)

Figure S2 **Aberrant organ development of cloned pigs.** (A) The vertical pathological dissection of the right side of the heart showed defects in CP2. An anatomically normal wild-type heart is shown in the left panel (WT heart). The CP2 heart exhibited aberrant valve development and pericarditis. The heart also showed right ventricular hypertrophy and heart hypoplasia. (B) The femur of CP1 was shorter and obviously mineralized in the epiphysis compared with a femur from a WT pig of the same age. The growth of the radius of CP1 was retarded compared with WT.
(TIF)

Table S1 **Raw data of** ***H19*** **putative DMR methylation percentages in different tissues of four cloned pigs and three wild-type pigs.**
(DOC)

Table S2 **Raw data of** ***IGF2*** **putative DMR methylation percentages in different tissues of four cloned pigs and three wild-type pigs.**
(DOC)

Table S3 **Raw data of** ***INS*** **putative DMR methylation percentages in four cloned pigs and three wild-type pigs.**
(DOC)

Table S4 **Raw data of** ***IGF2R*** **putative DMR methylation percentages in different tissues of four cloned pigs and three wild-type pigs.**
(DOC)

Table S5 **The methylation statuses of each imprinted gene in the analyzed cloned pig samples.**
(DOC)

Table S6 **The methylation statuses of each imprinted gene in all analyzed tissues of the four cloned pigs.**
(DOC)

Table S7 **The overall methylation patterns of each imprinted gene in all samples of cloned pigs.**
(DOC)

Table S8 **The percentage of aberrant methylation of the four imprinted genes in all analyzed tissues of the four cloned pigs.**
(DOC)
