# Supplementary figures and images for: Differential Differences in Methylation Status of Putative Imprinted Genes among Cloned Swine Genomes
Source: PLoS One. 2012 Feb 29;7(2):e32812. doi: 10.1371/journal.pone.0032812 (PMC3290620; doi:10.1371/journal.pone.0032812)

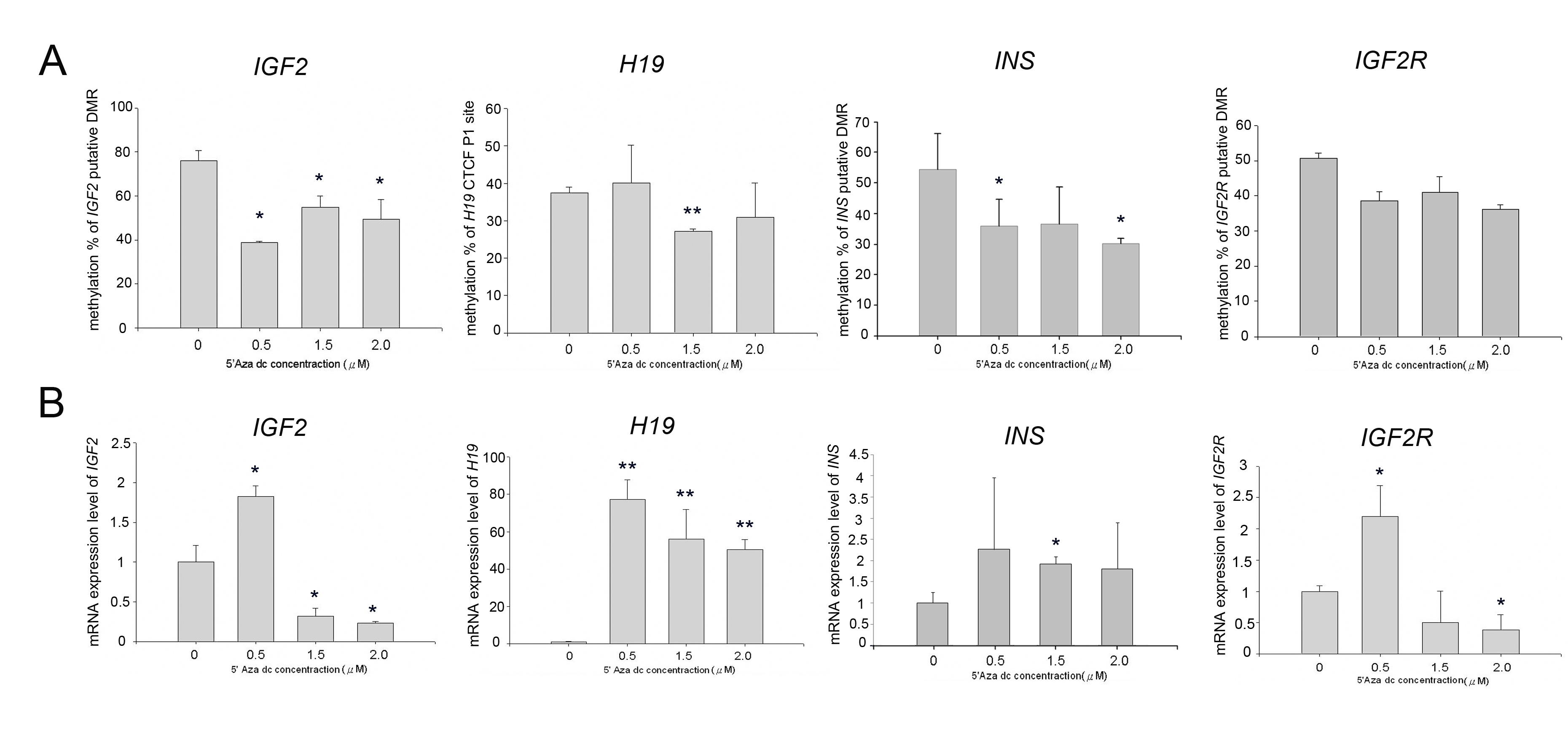

Supplement: Figure S1 — Changes in the methylation of putative DMRs and in the mRNA expression of four imprinted genes after treatment of pig ear fibroblasts with 5-aza-dc for 48 h. (A) The methylation percentage was quantified by COBRA. The methylation statuses of four imprinted genes (IGF2, H19, INS, and IGF2R) at their putative DMRs were decreased after different concentrations of 5-aza-dc treatment. (B) The mRNA expression was normalized to β-actin after real-time qRT-PCR. Three genes (IGF2, H19, and IGF2R) had increased mRNA expression after treatment with 0.5 µM 5-aza-dc. In contrast, the mRNA expression of INS significantly increased after treatment with 1.5 µM 5-aza-dc. The 5-aza-dc experiments were performed in 6-cm dishes seeded with 1.2×10^5^ pig fibroblasts in DMEM. All experiments were performed three times, and the data are expressed as the means ± SDs; *p<0.05, **p<0.01. (TIF) [file pone.0032812.s001.tif]

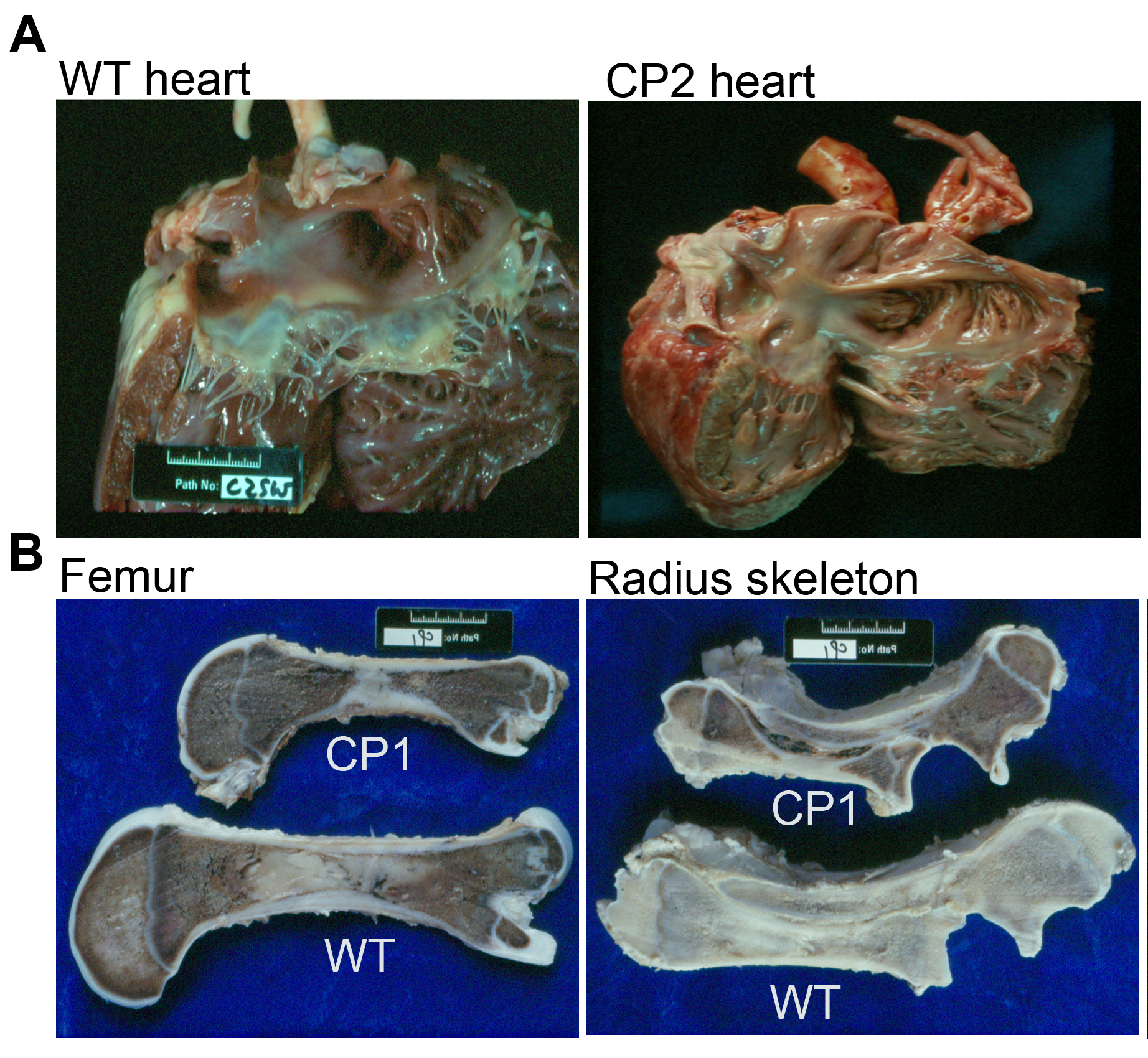

Supplement: Figure S2 — Aberrant organ development of cloned pigs. (A) The vertical pathological dissection of the right side of the heart showed defects in CP2. An anatomically normal wild-type heart is shown in the left panel (WT heart). The CP2 heart exhibited aberrant valve development and pericarditis. The heart also showed right ventricular hypertrophy and heart hypoplasia. (B) The femur of CP1 was shorter and obviously mineralized in the epiphysis compared with a femur from a WT pig of the same age. The growth of the radius of CP1 was retarded compared with WT. (TIF) [file pone.0032812.s002.tif]
