# Supplementary material for: Differential Differences in Methylation Status of Putative Imprinted Genes among Cloned Swine Genomes
Source: PLoS One. 2012 Feb 29;7(2):e32812. doi: 10.1371/journal.pone.0032812 (PMC3290620; doi:10.1371/journal.pone.0032812)
Supplement: Table S1 — Raw data of H19 putative DMR methylation percentages in different tissues of four cloned pigs and three wild-type pigs. (DOC) [file pone.0032812.s003.doc]

| **Table S1.** Raw data of *H19* putative DMR methylation percentages in different tissues of four cloned pigs and three wild-type pigs | | | | | | | | |
| --- | --- | --- | --- | --- | --- | --- | --- | --- |
| ***H19*** | **Mu** | **He** | **Ea** | **Li** | **Br** | **Lu** | **Ki** | **Pl** |
| **CP1** | **82.8** | nd | nd | **45.4** | 50.2 | 49.8 | **97.5** | **66.5** |
| **CP2** | **82.7** | **83.2** | **100** | **44.2** | nd | **68** | nd | **58.5** |
| **CP3** | **23.4** | **66.7** | **52** | 68.1 | nd | **56** | **0** | nd |
| **CP4** | **75.8** | nd | 63.7 | **0** | nd | nd | 44.2 | **61.6** |
| **WT1** | 65.7 | 59.8 | 64.7 | 62.5 | 35.8 | 46.6 | 51.8 | 25.6 |
| **WT2** | 52.9 | 54.2 | 67.6 | 63.9 | 43.6 | 38.6 | 41.2 | 38.3 |
| **WT3** | 46.7 | 46.3 | 62.2 | 54.2 | 41.6 | 36.6 | 40.3 | 45.1 |
| **Mean WT** | 55.1 | 53.4 | 64.8 | 60.2 | 40.3 | 40.6 | 44.4 | 36.3 |
| **±SD** | 9.7 | 6.8 | 2.7 | 5.2 | 4.1 | 5.3 | 6.4 | 9.9 |

Hypo- or hyper-methylation was defined as a ±10% change relative to the methylation percentage of WT tissue. Blue: cloned pigs; red: hypermethylation; green: hypomethylation; nd: not determined. CP1 sample size: 6; CP2 sample size: 6; CP3 sample size: 6; CP4 sample size: 5.
