# Supplementary material for: Differential Differences in Methylation Status of Putative Imprinted Genes among Cloned Swine Genomes
Source: PLoS One. 2012 Feb 29;7(2):e32812. doi: 10.1371/journal.pone.0032812 (PMC3290620; doi:10.1371/journal.pone.0032812)
Supplement: Table S2 — Raw data of IGF2 putative DMR methylation percentages in different tissues of four cloned pigs and three wild-type pigs. (DOC) [file pone.0032812.s004.doc]

**Table S2.** Raw data of *IGF2* putative DMR methylation percentages in different tissues of four cloned pigs and three wild-type pigs

| ***IGF2*** | **Mu** | **He** | **Ea** | **Li** | **Br** | **Lu** | **Ki** | **Pl** |
| --- | --- | --- | --- | --- | --- | --- | --- | --- |
| **CP1** | 73.5 | nd | nd | **75.3** | 80.4 | 70.6 | **52.6** | **43.2** |
| **CP2** | 72.1 | 88.2 | 69.7 | **86.1** | nd | 78.1 | nd | **54.6** |
| **CP3** | 57.6 | 89.9 | **85** | **67.8** | nd | **82.7** | **58.6** | nd |
| **CP4** | **84.8** | nd | 70.9 | 44 | nd | nd | 39.4 | **21.3** |
| **WT1** | 72.7 | 81.1 | 67.6 | 57.5 | 82.9 | 63.7 | 50.8 | 35.8 |
| **WT2** | 59.4 | 85.2 | 75.4 | 40.1 | 75.1 | 67.6 | 33.3 | 23.1 |
| **WT3** | 71.4 | 74.9 | 76.2 | 44 | 72.3 | 77.1 | 38.4 | 25.8 |
| **WT Mean** | 67.8 | 80.4 | 73.1 | 47.2 | 76.8 | 69.5 | 40.8 | 28.2 |
| **±SD** | 7.33 | 5.19 | 4.75 | 9.13 | 5.49 | 6.89 | 9.00 | 6.69 |

Hypo- or hyper-methylation was defined as a ±10% change relative to the methylation percentage of WT tissue. Blue: cloned pigs; red: hypermethylation; green: hypomethylation; nd: not determined. CP1 sample size: 6; CP2 sample size: 6; CP3 sample size: 6; CP4 sample size: 5.
