# Supplementary material for: Differential Differences in Methylation Status of Putative Imprinted Genes among Cloned Swine Genomes
Source: PLoS One. 2012 Feb 29;7(2):e32812. doi: 10.1371/journal.pone.0032812 (PMC3290620; doi:10.1371/journal.pone.0032812)
Supplement: Table S3 — Raw data of INS putative DMR methylation percentages in four cloned pigs and three wild-type pigs. (DOC) [file pone.0032812.s005.doc]

| **Table S3.** Raw data of *INS* putative DMR methylation percentages in four cloned pigs and three wild-type pigs | | | | | | | | |
| --- | --- | --- | --- | --- | --- | --- | --- | --- |
| ***INS*** | **Mu** | **He** | **Ea** | **Li** | **Br** | **Lu** | **Ki** | **Pl** |
| **CP1** | **62.1** | nd | nd | **70.2** | **80.9** | 54.1 | 47.4 | **99.4** |
| **CP2** | **58.8** | **70.8** | **77.1** | **78.1** | nd | 57.4 | nd | **58.6** |
| **CP3** | 41.9 | **77** | **99.5** | 58 | nd | 60.9 | 52.8 | nd |
| **CP4** | **17.4** | nd | **100** | 54.1 | nd | nd | 52 | 46.3 |
| **WT1** | 34.3 | 44.8 | 54.4 | 52.8 | 53.7 | 61.1 | 61.7 | 41 |
| **WT2** | 40.5 | 55.7 | 38.2 | 55.1 | 36 | 52.8 | 46.5 | 44.5 |
| **WT3** | 44.3 | 46.5 | 39.7 | 42.7 | 41.6 | 50.5 | 51 | 35 |
| **Mean WT** | 39.7 | 49.0 | 44.1 | 50.2 | 43.8 | 54.8 | 53.1 | 40.2 |
| **±SD** | 5.05 | 5.86 | 8.95 | 6.60 | 9.05 | 5.58 | 7.81 | 4.80 |

Hypo- or hyper-methylation was defined as a ±10% change relative to the methylation percentage of WT tissue. Blue: cloned pigs; red: hypermethylation; green: hypomethylation; nd: not determined. CP1 sample size: 6; CP2 sample size: 6; CP3 sample size: 6; CP4 sample size: 5.
