# Supplementary material for: Differential Differences in Methylation Status of Putative Imprinted Genes among Cloned Swine Genomes
Source: PLoS One. 2012 Feb 29;7(2):e32812. doi: 10.1371/journal.pone.0032812 (PMC3290620; doi:10.1371/journal.pone.0032812)
Supplement: Table S4 — Raw data of IGF2R putative DMR methylation percentages in different tissues of four cloned pigs and three wild-type pigs. (DOC) [file pone.0032812.s006.doc]

| **Table S4.** Raw data of *IGF2R* putative DMR methylation percentages in different tissues of four cloned pigs and three wild-type pigs | | | | | | | | |
| --- | --- | --- | --- | --- | --- | --- | --- | --- |
| ***IGF2R*** | **Mu** | **He** | **Ea** | **Li** | **Br** | **Lu** | **Ki** | **Pl** |
| **CP1** | **18.9** | nd | nd | **47.2** | 64.4 | **31.3** | 24.1 | 39.6 |
| **CP2** | 29.7 | **24.8** | 35.7 | **52.8** | nd | **34.7** | nd | 22.4 |
| **CP3** | 33.7 | **26.9** | 42.2 | **14.6** | nd | 45.9 | 27.3 | nd |
| **CP4** | **41.7** | nd | **32.5** | **18** | nd | nd | **8.7** | 34.7 |
| **WT1** | 24.4 | 46.4 | 32.6 | 34.8 | 66.9 | 54.5 | 24.1 | 24.5 |
| **WT2** | 28.9 | 42 | 49 | 34.2 | 77.8 | 51.8 | 24.8 | 26.9 |
| **WT3** | 40 | 41.2 | 47.3 | 27.8 | 85.6 | 57.2 | 26.6 | 40.3 |
| **Mean WT** | 31.1 | 43.2 | 43.0 | 32.3 | 76.8 | 54.5 | 25.2 | 30.6 |
| **±SD** | 8.03 | 2.80 | 9.02 | 3.88 | 9.39 | 2.70 | 1.29 | 8.51 |

Hypo- or hyper-methylation was defined as a ±10% change relative to the methylation percentage of WT tissue. Blue: cloned pigs; red: hypermethylation; green: hypomethylation; nd: not determined. CP1 sample size: 6; CP2 sample size: 6; CP3 sample size: 6; CP4 sample size: 5.
