# Supplementary material for: Differential Differences in Methylation Status of Putative Imprinted Genes among Cloned Swine Genomes
Source: PLoS One. 2012 Feb 29;7(2):e32812. doi: 10.1371/journal.pone.0032812 (PMC3290620; doi:10.1371/journal.pone.0032812)
Supplement: Table S5 — The methylation statuses of each imprinted gene in the analyzed cloned pig samples. (DOC) [file pone.0032812.s007.doc]

**Table S5.** The methylation statuses of each imprinted gene in the analyzed cloned pig samples

| Genes | Hypermethylation (%) | Normal pattern (%) | Hypomethylation (%) |
| --- | --- | --- | --- |
| *H19* | 9/20 (45) | 5/20 (25) | 6/20 (30) |
| *IGF2* | 8/20 (40) | 12/20 (60) | 0/20 (0) |
| *INS* | 10/20 (50) | 9/20 (45) | 1/20 (5) |
| *IGF2R* | 3/20 (15) | 8/20 (40) | 9/20 (45) |
