# Supplementary material for: Differential Differences in Methylation Status of Putative Imprinted Genes among Cloned Swine Genomes
Source: PLoS One. 2012 Feb 29;7(2):e32812. doi: 10.1371/journal.pone.0032812 (PMC3290620; doi:10.1371/journal.pone.0032812)
Supplement: Table S6 — The methylation statuses of each imprinted gene in all analyzed tissues of the four cloned pigs. (DOC) [file pone.0032812.s008.doc]

**Table S6.** The methylation statuses of each imprinted gene in all analyzed tissues of the four cloned pigs

|  | Total | P^b^ | M^c^ | *H19* | *IGF2R* | *IGF2* | *INS* |
| --- | --- | --- | --- | --- | --- | --- | --- |
| CP1 | 11/20 (55)^a^ | 6/10 (60) | 5/10 (50) | 3/5 (60) | 3/5 (60) | 2/5 (40) | 3/5 (60) |
| CP2 | 13/20 (65) | 8/10 (80) | 5/10 (50) | 5/5 (100) | 3/5 (60) | 1/5 (20) | 4/5 (80) |
| CP3 | 13/20 (65) | 7/12 (58) | 6/12 (50) | 5/6 (83) | 2/6 (33) | 4/6 (67) | 2/6 (33) |
| CP4 | 9/16 (56) | 6/8 (75) | 2/8 (25) | 2/4 (50) | 4/4 (100) | 1/4 (25) | 2/4 (50) |

^a^The number is presented as total aberrant samples / analyzed samples (%); ^b^M: maternally imprinted genes (*H19*

and *IGF2R*); ^c^P: paternally imprinted genes (*IGF2* and *INS*).
