# Supplementary material for: Differential Differences in Methylation Status of Putative Imprinted Genes among Cloned Swine Genomes
Source: PLoS One. 2012 Feb 29;7(2):e32812. doi: 10.1371/journal.pone.0032812 (PMC3290620; doi:10.1371/journal.pone.0032812)
Supplement: Table S7 — The overall methylation patterns of each imprinted gene in all samples of cloned pigs. (DOC) [file pone.0032812.s009.doc]

**Table S7. The overall methylation patterns of each imprinted gene in all samples of cloned pigs**

|  |  | *H19* |  | *IGF2R* | | |  | *IGF2* |  |  | *INS* |  |
| --- | --- | --- | --- | --- | --- | --- | --- | --- | --- | --- | --- | --- |
|  | hyper-^b^ | normal | hypo-^c^ | hyper- | normal | hypo- | hyper- | normal | hypo- | hyper- | normal | hypo- |
| CP1 | 2/5 (40)^a^ | 2/5 (40) | 1/5 (20) | 1/5 (20) | 2/5 (40) | 2/5 (40) | 2/5 (40) | 3/5 (60) | 0/5 (0) | 3/5 (60) | 2/5 (40) | 0/5 (0) |
| CP2 | 4/5 (80) | 0/5 (0) | 1/5 (20) | 1/5 (20) | 2/5 (40) | 2/5 (40) | 1/5 (20) | 4/5 (80) | 0/5 (0) | 4/5 (80) | 1/5 (20) | 0/5 (0) |
| CP3 | 2/6 (33) | 1/6 (17) | 3/6 (50) | 0/6 (0) | 4/6 (67) | 2/6 (33) | 4/6 (67) | 2/6 (33) | 0/6 (0) | 2/6 (33) | 4/6 (67) | 0/6 (0) |
| CP4 | 1/4 (25) | 2/4 (50) | 1/4 (25) | 1/4 (25) | 0/4 (0) | 3/4 (75) | 1/4 (25) | 3/4 (75) | 0/4 (0) | 1/4 (25) | 2/4 (50) | 1/4 (25) |

^a^The number is presented as total aberrant samples / analyzed samples (%); ^b^hyper-: hypermethylation pattern;

^c^hypo-: hypomethylation pattern.
