# Supplementary material for: Differential Differences in Methylation Status of Putative Imprinted Genes among Cloned Swine Genomes
Source: PLoS One. 2012 Feb 29;7(2):e32812. doi: 10.1371/journal.pone.0032812 (PMC3290620; doi:10.1371/journal.pone.0032812)
Supplement: Table S8 — The percentage of aberrant methylation of the four imprinted genes in all analyzed tissues of the four cloned pigs. (DOC) [file pone.0032812.s010.doc]

**Table S8.** The percentage of aberrant methylation of the four imprinted genes in all analyzed tissues of the four cloned pigs

| Cloned pigs | Hypermethylation (%) | Normal pattern (%) | Hypomethylation (%) |
| --- | --- | --- | --- |
| CP1 | 8/20 (40) | 9/20 (45) | 3/20 (15) |
| CP2 | 10/20 (50) | 7/20 (35) | 3/20 (15) |
| CP3 | 8/24 (33) | 11/24 (46) | 5/24 (21) |
| CP4 | 4/16 (25) | 5/16 (31) | 7/16 (44) |
